# 3-(4-Fluoro­phen­yl)-2-(4-pyrid­yl)pyrido[2,3-*b*]pyrazine

**DOI:** 10.1107/S1600536809037970

**Published:** 2009-09-26

**Authors:** Pierre Koch, Dieter Schollmeyer, Stefan Laufer

**Affiliations:** a Institute of Pharmacy, Department of Pharmaceutical and Medicinal Chemistry, Eberhard-Karls-University Tübingen, Auf der Morgenstelle 8, 72076 Tübingen, Germany; b Department of Organic Chemistry, Johannes Gutenberg-University Mainz, Duesbergweg 10-14, D-55099 Mainz, Germany

## Abstract

In the crystal structure of the title compound, C_18_H_11_FN_4_, the pyridopyrazine ring makes dihedral angles of 34.67 (7) and 52.24 (7)° with the 4-fluoro­phenyl and pyridine rings, respectively. The 4-fluoro­phenyl ring makes a dihedral angle of 59.56 (9)° with the pyridine ring.

## Related literature

For preparation of pyridopyrazines under microwave conditions, see: Zhao *et al*. (2004 ▶).
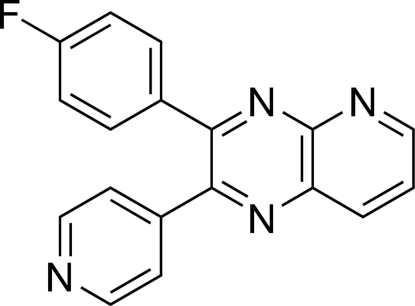


## Experimental

### 

#### Crystal data

C_18_H_11_FN_4_
*M* *_r_* = 302.31
Monoclinic, 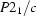

*a* = 9.7163 (9) Å
*b* = 13.7937 (6) Å
*c* = 10.8164 (10) Å
β = 90.994 (5)°
*V* = 1449.4 (2) Å^3^
*Z* = 4
Cu *K*α radiation
μ = 0.78 mm^−1^
*T* = 193 K
0.30 × 0.25 × 0.22 mm

#### Data collection

Enraf–Nonius CAD-4 diffractometer
Absorption correction: none
2906 measured reflections
2753 independent reflections
2654 reflections with *I* > 2σ(*I*)
*R*_int_ = 0.023
3 standard reflections frequency: 60 min intensity decay: 2%

#### Refinement

*R*[*F* ^2^ > 2σ(*F* ^2^)] = 0.047
*wR*(*F* ^2^) = 0.149
*S* = 1.20
2753 reflections
209 parameters
H-atom parameters constrained
Δρ_max_ = 0.31 e Å^−3^
Δρ_min_ = −0.24 e Å^−3^

### 

Data collection: *CAD-4 Software* (Enraf–Nonius, 1989 ▶); cell refinement: *CAD-4 Software*; data reduction: *CORINC* (Dräger & Gattow, 1971 ▶); program(s) used to solve structure: *SIR97* (Altomare *et al.*, 1999 ▶); program(s) used to refine structure: *SHELXL97* (Sheldrick, 2008 ▶); molecular graphics: *PLATON* (Spek, 2009 ▶); software used to prepare material for publication: *PLATON*.

## Supplementary Material

Crystal structure: contains datablocks I, global. DOI: 10.1107/S1600536809037970/bt5066sup1.cif

Structure factors: contains datablocks I. DOI: 10.1107/S1600536809037970/bt5066Isup2.hkl

Additional supplementary materials:  crystallographic information; 3D view; checkCIF report

## Figures and Tables

**Fig. 1.**
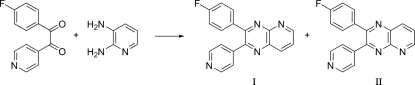
Synthesis of I and II.

**Fig. 2.**
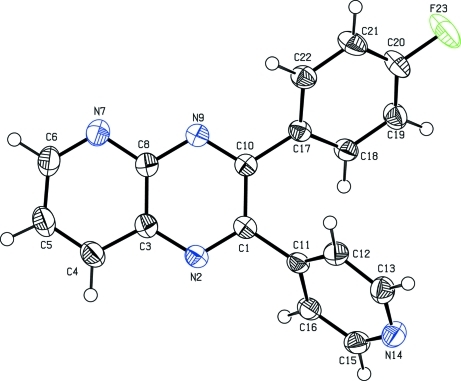
View of compound I. Displacement ellipsoids are drawn at the 50% probability level.

## supplementary crystallographic information

### Comment

The title compound, 3-(4-fluorophenyl)-2-(pyridin-4-yl)pyrido[2,3-*b*]pyrazine (I), was prepared in the course of our studies on pyridin-4-yl-substituted pyridopyrazines as p38 mitogen-activated protein (MAP) kinase inhibitors.

The microwave-assisted reaction of 1-(4-fluorophenyl)-2-(pyridin-4-yl)ethane-1,2-dione and 2,3-diaminopyridine yields two regioisomers, 3-(4-fluorophenyl)-2-(pyridin-4-yl)pyrido[2,3-*b*]pyrazine (I) and 2-(4-fluorophenyl)-3-(pyridin-4-yl)pyrido[3,2-*b*]pyrazine (II) (Figure I). The isomers were separated by flash-chromatography. To identify the two regioisomers *x*-ray analysis was used. In this article we present the X-ray data of the first eluted isomer I.

As might be expected the 4-fluorophenyl, the pyridine ring as well as the pyridopyrazine ring are planar (Figure 2). The pyridopyrazine ring makes dihedral angles of 34.67 (7)° and 52.24 (7)° to the 4-fluorophenyl ring and the pyridine ring, respectively. The 4-fluorophenyl ring makes a dihedral angle of 59.56 (9)° to the pyridine ring.

### Experimental

1-(4-Fluorophenyl)-2-(pyridin-4-yl)ethane-1,2-dione (113 mg, 0.5 mmol), and 2,3-diaminopyridine (54 mg, 0.5 mmol), and methanol/glacial acetic acid (2 ml, 9:1, V:V) were combined in a reaction vial. The reaction vessel was heated in a microwave reactor for 5 min at 433 K (initial power 250 W), after which a stream of compressed air cooled the reaction vessel to r.t. The solvent was removed under reduced pressure and the residue was purified by flash-chromatography (silica gel, petroleum ether/ethyl acetate 1–4 to 0–1) to yield 67 mg (44%) of I as a colorless solid. Suitable crystals of compound I for X-ray were obtained by slow evaporation at 298 K of a solution of n-hexane - diethyl ether (2–1).

### Refinement

Hydrogen atoms   were placed at calculated positions with C—H = 0.95 Å . They were refined in the riding-model approximation with isotropic displacement parameters  set at 1.2 times of the *U*_eq_ of the parent atom.

### Crystal data

**Table:**

C_18_H_11_FN_4_	*F*(000) = 624
*M**_r_* = 302.31	*D*_x_ = 1.385 Mg m^−^^3^
Monoclinic, *P*2_1_/*c*	Cu *K*α radiation, λ = 1.54178 Å
Hall symbol: -P 2ybc	Cell parameters from 25 reflections
*a* = 9.7163 (9) Å	θ = 65–69°
*b* = 13.7937 (6) Å	µ = 0.78 mm^−^^1^
*c* = 10.8164 (10) Å	*T* = 193 K
β = 90.994 (5)°	Block, colourless
*V* = 1449.4 (2) Å^3^	0.30 × 0.25 × 0.22 mm
*Z* = 4	

### Data collection

**Table:**

Enraf–Nonius CAD-4 diffractometer	*R*_int_ = 0.023
Radiation source: rotating anode	θ_max_ = 70.0°, θ_min_ = 4.6°
graphite	*h* = −11→11
ω/2θ scans	*k* = 0→16
2906 measured reflections	*l* = 0→13
2753 independent reflections	3 standard reflections every 60 min
2654 reflections with *I* > 2σ(*I*)	intensity decay: 2%

### Refinement

**Table:**

Refinement on *F*^2^	Secondary atom site location: difference Fourier map
Least-squares matrix: full	Hydrogen site location: inferred from neighbouring sites
*R*[*F*^2^ > 2σ(*F*^2^)] = 0.047	H-atom parameters constrained
*wR*(*F*^2^) = 0.149	*w* = 1/[σ^2^(*F*_o_^2^) + (0.073*P*)^2^ + 0.6448*P*] where *P* = (*F*_o_^2^ + 2*F*_c_^2^)/3
*S* = 1.20	(Δ/σ)_max_ < 0.001
2753 reflections	Δρ_max_ = 0.31 e Å^−^^3^
209 parameters	Δρ_min_ = −0.24 e Å^−^^3^
0 restraints	Extinction correction: *SHELXL97* (Sheldrick, 2008), Fc^*^=kFc[1+0.001xFc^2^λ^3^/sin(2θ)]^-1/4^
Primary atom site location: structure-invariant direct methods	Extinction coefficient: 0.0079 (9)

### Special details

**Table:**

Geometry. All e.s.d.'s (except the e.s.d. in the dihedral angle between two l.s. planes) are estimated using the full covariance matrix. The cell e.s.d.'s are taken into account individually in the estimation of e.s.d.'s in distances, angles and torsion angles; correlations between e.s.d.'s in cell parameters are only used when they are defined by crystal symmetry. An approximate (isotropic) treatment of cell e.s.d.'s is used for estimating e.s.d.'s involving l.s. planes.
Refinement. Refinement of *F*^2^ against ALL reflections. The weighted *R*-factor *wR* and goodness of fit *S* are based on *F*^2^, conventional *R*-factors *R* are based on *F*, with *F* set to zero for negative *F*^2^. The threshold expression of *F*^2^ > σ(*F*^2^) is used only for calculating *R*-factors(gt) *etc*. and is not relevant to the choice of reflections for refinement. *R*-factors based on *F*^2^ are statistically about twice as large as those based on *F*, and *R*- factors based on ALL data will be even larger.

### Fractional atomic coordinates and isotropic or equivalent isotropic displacement parameters (Å^2^)

**Table:**

	*x*	*y*	*z*	*U*_iso_*/*U*_eq_	
C1	0.71724 (16)	0.38396 (12)	0.46833 (15)	0.0271 (4)	
N2	0.82823 (14)	0.41887 (10)	0.52355 (13)	0.0300 (4)	
C3	0.81675 (18)	0.44950 (12)	0.64285 (16)	0.0294 (4)	
C4	0.9322 (2)	0.48632 (15)	0.70813 (17)	0.0383 (4)	
H4	1.0203	0.4880	0.6715	0.046*	
C5	0.9135 (2)	0.51935 (15)	0.82524 (18)	0.0430 (5)	
H5	0.9892	0.5436	0.8727	0.052*	
C6	0.7806 (2)	0.51712 (15)	0.87511 (18)	0.0422 (5)	
H6	0.7698	0.5425	0.9560	0.051*	
N7	0.67047 (17)	0.48270 (12)	0.81831 (14)	0.0385 (4)	
C8	0.68851 (18)	0.44723 (12)	0.70263 (15)	0.0289 (4)	
N9	0.57616 (14)	0.40774 (11)	0.64607 (13)	0.0297 (4)	
C10	0.58915 (16)	0.37376 (11)	0.53266 (15)	0.0263 (4)	
C11	0.73166 (16)	0.36165 (12)	0.33454 (15)	0.0277 (4)	
C12	0.64457 (18)	0.40275 (13)	0.24579 (16)	0.0324 (4)	
H12	0.5701	0.4429	0.2695	0.039*	
C13	0.6681 (2)	0.38414 (14)	0.12271 (17)	0.0377 (4)	
H13	0.6082	0.4129	0.0629	0.045*	
N14	0.77022 (17)	0.32808 (12)	0.08247 (15)	0.0406 (4)	
C15	0.85276 (19)	0.28932 (14)	0.16925 (18)	0.0370 (4)	
H15	0.9263	0.2494	0.1428	0.044*	
C16	0.83866 (17)	0.30326 (13)	0.29441 (17)	0.0322 (4)	
H16	0.9005	0.2737	0.3520	0.039*	
C17	0.46722 (16)	0.32309 (12)	0.47894 (15)	0.0270 (4)	
C18	0.48092 (17)	0.24153 (13)	0.40375 (16)	0.0307 (4)	
H18	0.5702	0.2194	0.3830	0.037*	
C19	0.36587 (19)	0.19234 (14)	0.35891 (18)	0.0363 (4)	
H19	0.3750	0.1363	0.3086	0.044*	
C20	0.23859 (18)	0.22707 (15)	0.3895 (2)	0.0405 (5)	
C21	0.21958 (18)	0.30696 (14)	0.4637 (2)	0.0404 (5)	
H21	0.1298	0.3289	0.4831	0.049*	
C22	0.33549 (17)	0.35415 (13)	0.50905 (17)	0.0329 (4)	
H22	0.3251	0.4087	0.5617	0.039*	
F23	0.12564 (12)	0.17883 (11)	0.34584 (16)	0.0658 (5)	

### Atomic displacement parameters (Å^2^)

**Table:**

	*U*^11^	*U*^22^	*U*^33^	*U*^12^	*U*^13^	*U*^23^
C1	0.0255 (8)	0.0238 (8)	0.0320 (9)	0.0011 (6)	−0.0009 (6)	0.0019 (6)
N2	0.0273 (7)	0.0305 (7)	0.0322 (7)	−0.0026 (6)	0.0003 (6)	0.0008 (6)
C3	0.0308 (9)	0.0265 (8)	0.0307 (8)	−0.0022 (6)	−0.0022 (7)	0.0029 (6)
C4	0.0338 (9)	0.0418 (10)	0.0390 (10)	−0.0094 (8)	−0.0035 (7)	0.0022 (8)
C5	0.0460 (11)	0.0450 (11)	0.0375 (10)	−0.0158 (9)	−0.0105 (8)	0.0024 (8)
C6	0.0540 (12)	0.0420 (11)	0.0304 (9)	−0.0102 (9)	−0.0038 (8)	−0.0039 (8)
N7	0.0420 (9)	0.0417 (9)	0.0320 (8)	−0.0044 (7)	0.0009 (6)	−0.0040 (6)
C8	0.0314 (8)	0.0258 (8)	0.0295 (8)	−0.0006 (6)	−0.0011 (6)	0.0018 (6)
N9	0.0283 (7)	0.0307 (8)	0.0303 (7)	−0.0004 (6)	0.0007 (5)	−0.0001 (6)
C10	0.0263 (8)	0.0236 (8)	0.0290 (8)	0.0019 (6)	−0.0001 (6)	0.0008 (6)
C11	0.0243 (8)	0.0273 (8)	0.0315 (9)	−0.0045 (6)	0.0026 (6)	−0.0010 (6)
C12	0.0319 (9)	0.0315 (9)	0.0338 (9)	0.0008 (7)	0.0023 (7)	0.0009 (7)
C13	0.0413 (10)	0.0387 (10)	0.0329 (9)	−0.0040 (8)	−0.0023 (7)	0.0012 (7)
N14	0.0454 (9)	0.0425 (9)	0.0341 (8)	−0.0067 (7)	0.0062 (7)	−0.0070 (7)
C15	0.0324 (9)	0.0349 (9)	0.0441 (10)	−0.0043 (7)	0.0090 (8)	−0.0096 (8)
C16	0.0254 (8)	0.0327 (9)	0.0384 (9)	−0.0022 (7)	0.0012 (7)	−0.0041 (7)
C17	0.0246 (8)	0.0279 (8)	0.0283 (8)	−0.0010 (6)	0.0000 (6)	0.0035 (6)
C18	0.0251 (8)	0.0315 (9)	0.0356 (9)	−0.0002 (7)	0.0010 (6)	−0.0007 (7)
C19	0.0341 (9)	0.0330 (9)	0.0418 (10)	−0.0029 (7)	−0.0028 (7)	−0.0063 (7)
C20	0.0263 (9)	0.0387 (10)	0.0561 (12)	−0.0063 (7)	−0.0085 (8)	−0.0023 (9)
C21	0.0235 (9)	0.0386 (10)	0.0593 (12)	0.0021 (7)	0.0004 (8)	−0.0008 (9)
C22	0.0285 (9)	0.0295 (9)	0.0407 (9)	0.0020 (7)	0.0024 (7)	−0.0014 (7)
F23	0.0305 (6)	0.0594 (9)	0.1070 (12)	−0.0084 (6)	−0.0157 (7)	−0.0256 (8)

### Geometric parameters (Å, °)

**Table:**

C1—N2	1.315 (2)	C12—H12	0.9500
C1—C10	1.443 (2)	C13—N14	1.336 (3)
C1—C11	1.488 (2)	C13—H13	0.9500
N2—C3	1.364 (2)	N14—C15	1.336 (3)
C3—C4	1.410 (2)	C15—C16	1.377 (3)
C3—C8	1.414 (2)	C15—H15	0.9500
C4—C5	1.361 (3)	C16—H16	0.9500
C4—H4	0.9500	C17—C22	1.394 (2)
C5—C6	1.409 (3)	C17—C18	1.396 (2)
C5—H5	0.9500	C18—C19	1.388 (2)
C6—N7	1.313 (2)	C18—H18	0.9500
C6—H6	0.9500	C19—C20	1.372 (3)
N7—C8	1.358 (2)	C19—H19	0.9500
C8—N9	1.356 (2)	C20—F23	1.361 (2)
N9—C10	1.321 (2)	C20—C21	1.378 (3)
C10—C17	1.485 (2)	C21—C22	1.383 (3)
C11—C12	1.389 (2)	C21—H21	0.9500
C11—C16	1.391 (2)	C22—H22	0.9500
C12—C13	1.379 (3)		
			
N2—C1—C10	121.60 (15)	C11—C12—H12	120.6
N2—C1—C11	115.28 (14)	N14—C13—C12	123.97 (18)
C10—C1—C11	123.03 (14)	N14—C13—H13	118.0
C1—N2—C3	117.59 (14)	C12—C13—H13	118.0
N2—C3—C4	120.53 (16)	C15—N14—C13	116.28 (16)
N2—C3—C8	120.86 (15)	N14—C15—C16	124.50 (17)
C4—C3—C8	118.60 (16)	N14—C15—H15	117.7
C5—C4—C3	117.99 (17)	C16—C15—H15	117.7
C5—C4—H4	121.0	C15—C16—C11	118.40 (17)
C3—C4—H4	121.0	C15—C16—H16	120.8
C4—C5—C6	119.12 (17)	C11—C16—H16	120.8
C4—C5—H5	120.4	C22—C17—C18	118.76 (15)
C6—C5—H5	120.4	C22—C17—C10	119.57 (15)
N7—C6—C5	125.01 (18)	C18—C17—C10	121.58 (15)
N7—C6—H6	117.5	C19—C18—C17	120.87 (16)
C5—C6—H6	117.5	C19—C18—H18	119.6
C6—N7—C8	116.37 (16)	C17—C18—H18	119.6
N9—C8—N7	116.40 (15)	C20—C19—C18	117.97 (17)
N9—C8—C3	120.75 (15)	C20—C19—H19	121.0
N7—C8—C3	122.84 (16)	C18—C19—H19	121.0
C10—N9—C8	118.18 (14)	F23—C20—C19	118.07 (18)
N9—C10—C1	120.67 (15)	F23—C20—C21	118.54 (17)
N9—C10—C17	116.22 (14)	C19—C20—C21	123.38 (17)
C1—C10—C17	123.09 (14)	C20—C21—C22	117.80 (17)
C12—C11—C16	118.06 (16)	C20—C21—H21	121.1
C12—C11—C1	121.39 (15)	C22—C21—H21	121.1
C16—C11—C1	120.45 (15)	C21—C22—C17	121.19 (17)
C13—C12—C11	118.80 (17)	C21—C22—H22	119.4
C13—C12—H12	120.6	C17—C22—H22	119.4
			
C10—C1—N2—C3	−2.9 (2)	N2—C1—C11—C16	52.5 (2)
C11—C1—N2—C3	173.66 (14)	C10—C1—C11—C16	−130.92 (17)
C1—N2—C3—C4	179.03 (16)	C16—C11—C12—C13	−0.2 (2)
C1—N2—C3—C8	−2.5 (2)	C1—C11—C12—C13	176.03 (16)
N2—C3—C4—C5	177.47 (17)	C11—C12—C13—N14	0.3 (3)
C8—C3—C4—C5	−1.0 (3)	C12—C13—N14—C15	−0.3 (3)
C3—C4—C5—C6	−1.2 (3)	C13—N14—C15—C16	0.2 (3)
C4—C5—C6—N7	1.9 (3)	N14—C15—C16—C11	−0.1 (3)
C5—C6—N7—C8	−0.2 (3)	C12—C11—C16—C15	0.1 (2)
C6—N7—C8—N9	177.15 (16)	C1—C11—C16—C15	−176.17 (15)
C6—N7—C8—C3	−2.2 (3)	N9—C10—C17—C22	34.0 (2)
N2—C3—C8—N9	5.1 (2)	C1—C10—C17—C22	−147.77 (17)
C4—C3—C8—N9	−176.46 (16)	N9—C10—C17—C18	−142.59 (16)
N2—C3—C8—N7	−175.65 (16)	C1—C10—C17—C18	35.6 (2)
C4—C3—C8—N7	2.8 (3)	C22—C17—C18—C19	0.4 (3)
N7—C8—N9—C10	179.00 (15)	C10—C17—C18—C19	177.00 (16)
C3—C8—N9—C10	−1.7 (2)	C17—C18—C19—C20	0.8 (3)
C8—N9—C10—C1	−3.8 (2)	C18—C19—C20—F23	−179.86 (17)
C8—N9—C10—C17	174.53 (14)	C18—C19—C20—C21	−1.1 (3)
N2—C1—C10—N9	6.4 (2)	F23—C20—C21—C22	178.90 (18)
C11—C1—C10—N9	−169.95 (15)	C19—C20—C21—C22	0.1 (3)
N2—C1—C10—C17	−171.78 (15)	C20—C21—C22—C17	1.1 (3)
C11—C1—C10—C17	11.9 (2)	C18—C17—C22—C21	−1.4 (3)
N2—C1—C11—C12	−123.57 (17)	C10—C17—C22—C21	−178.07 (16)
C10—C1—C11—C12	53.0 (2)		
